# Impact of the COVID-19 pandemic on Canadian performing and creative artists: An interpretive descriptive study using the social-ecological model

**DOI:** 10.1371/journal.pone.0310369

**Published:** 2024-09-17

**Authors:** Shelly-Anne Li, Clive Stevens, Coco Zhang Ke Jiang

**Affiliations:** 1 Department of Family & Community Medicine, Temerty Faculty of Medicine, University of Toronto, Toronto, Ontario, Canada; 2 The Al & Malka Green Artists’ Health Centre, University Health Network, Toronto, Ontario, Canada; Chulalongkorn University Faculty of Medicine, THAILAND

## Abstract

**Background:**

Public health restrictions during the Coronavirus-2019 (COVID-19) pandemic in Canada have substantially reduced the work and income of performing and creative artists. We aimed to understand how factors at the public policy, community, organizational, interpersonal and individual levels affected Canadian performing and creative artists’ health and livelihood during the pandemic.

**Methods:**

We interviewed 14 creative and performing artists from an academic hospital-based healthcare center in Toronto, Canada. In addition, we conducted secondary data analysis on an existing set of 17 transcribed interviews from a quality improvement study that included relevant information to answer the present study’s research question. We applied an interpretive descriptive approach to our qualitative inquiry and used the social-ecological model (SEM) as our analytic framework.

**Results:**

We identified factors at all levels of the SEM that tended to synergistically affect the health and livelihood of artists during the COVID-19 pandemic. Public health restrictions and government financial assistance programs have downstream effects on other levels. During the pandemic, many artists sensed an overwhelming loss of community, financial instability, and limited access to healthcare; which in turn affected their health. For those who accessed financial assistance programs, the stability of income afforded time for rest without the stress of food insecurity or housing instability.

**Conclusions:**

Use of the SEM as an analytic framework reflects the multidirectional intricacy and dynamic interplay among factors operating within and across all five levels, bringing to light potential areas of improvement at various levels to strengthen resilience and reduce risk factors associated with artists’ health and healthcare access. Findings also accentuated the fragility of precarious work that inundates the performing arts industry, which emphasizes the need for interventions and policies to address this issue. Such interventions might include financial support programs for artists, access to affordable healthcare services, and efforts to strengthen social support networks within the arts community.

## Introduction

Canadian creative and performing artists lost a total of over 20 million CAD in income, which represents an average of 83% in reduced income per artist due to the coronavirus disease 2019 (COVID-19) pandemic [1]. As the pandemic persisted, one in two artists have permanently lost work by the end of 2021 [2, 3]. Canada experienced one of the world’s longest lockdowns (>360 days) since the start of the pandemic in March 2020. Physical distancing and restrictions on social and public gathering were intended to reduce COVID-19 transmission. Subsequently, these restrictions led to the cancellation or postponement of performances and showings across all Canadian provinces, which substantially limited operations of arts organizations. Even with growing digital sales, government grants, and subsidies aimed at helping arts businesses pay arts workers and artists; the performing and creative arts industry revenues declined by a record of 1.8 billion CAD in 2020 [4]. After the airline industry, the arts, entertainment and recreation sector represents the hardest-hit area of the economy, accounting for over 10% of all job losses in Canada [5].

The Government of Canada classifies creative and performing artists under a single job category that includes producers, directors, choreographers, conductors, acrobats, composers, musicians, arts workers, photographers, singers, dancers, actors, comedians, painters, sculptors and other visual artists [6]. Long before COVID-19, creative and performing artists often experienced income volatility and healthcare insecurity. The chronic lack of access to affordable, employer-subsidized healthcare for freelance artists, which make up 65% of all Canadian artists, has created an even greater vulnerability and financial burden during the pandemic in this group [5]. The pandemic is occurring against the backdrop of the already heightened prevalence of occupational stress and mental health issues among creative and performing artists. The implications of COVID-19 are associated with an exacerbation of pre-existing mental health issues, greater difficulty in accessing mental health support and services, and greater financial insecurity [7, 8]. Performing artists, who have typically earned lower income and experienced greater income volatility compared to creative artists before the pandemic [5], were particularly impacted by public health restrictions during the pandemic; 96% reported feeling stressed about their occupation and reduced income. In this category, over 70% reported feeling either very stressed or extremely stressed [1].

Public health restrictions imposed by governments have disrupted the arts industry across nations; the closure of performance spaces and the cancellation of events led to the loss of income and the limited opportunities for collaboration. These restrictions have had a cascading effect on their mental and physical health, as well as on their ability to maintain social connections and participate in creative activities. One survey study identified five main effects that public health restrictions have had on performing artists in the United Kingdom, including: financial concerns and job uncertainties; challenges of transitioning to online work, reduced social connections and lack of support, and poorer mental and physical health (e.g., anxiety, lack of motivation) [9].

Bronfenbrenner’s social-ecological model (SEM) provides a useful framework for investigating the multiple levels of influence that affect the health and healthcare access of artists. This model recognizes that individuals are shaped by and interact with their immediate social environment, which is in turn shaped by and interacts with the broader public policy, organizational, and community contexts. Applying this model allows us to understand the complex interplay that influence the health and well-being of artists, including factors such as financial stability, mental health, social support, access to resources, and public health restrictions. Artists operate in multiple environments (e.g., workplace, neighborhood, professional communities) that “spill over” and influence each other. The same environment may have different effects on an artist’s health depending on a variety of factors that reside in other levels of the SEM.

Although published studies have demonstrated associations between the pandemic lockdown and artists’ health status [9–12], less is known about exactly how these lockdowns have impacted their health and healthcare access. This qualitative study aims to understand the impact of the COVID-19 pandemic on the health and livelihood of Canadian artists.

## Methods

We applied interpretive description to gain an in-depthunderstanding of the subjective experiences and meanings of creative and performing artists whose health and wellbeing have been impacted by the COVID-19 pandemic. Interpretive description allows for in-depth exploration of the experiences, perspectives and meanings using the SEM as an analytic framework. The goal in using this approach was to identify themes and patterns across the individual interviews, and interpret the meaning of these themes using the SEM. We received a waiver from the Research Ethics Board of University Health Network (ID# 22–0348) because the study was deemed as a quality improvement project; findings of this study were summarized as recommendations to inform program development and funding allocation to healthcare services at the Centre. Participants of the transcripts that were used for secondary data analysis also provided consent to allowing subsequent analysis on their data at their time of interview. At the time of reporting, recommendations (e.g., monthly facilitator-led, virtual spaces for artists to build a sense of community) have been implemented.

### Setting

We recruited creative and performing artists between February and May 2022 from The Al & Malka Green Artists’ Health Centre, a university-affiliated, hospital-based healthcare centre located in Toronto, a large metropolitan city in Canada. The Centre provides specialized healthcare services to almost 2000 artists of all disciplines. The Centre provides complementary healthcare services including chiropractic services, psychotherapy, counselling, massage therapy, shiatsu therapy, acupuncture, and naturopathic medicine. Artists who visit for the first time do an initial intake assessment with a nurse coordinator, who provides an overall assessment of the artist and requests for tests and do referrals to other specialized health services that would be covered by the Ontario Health Insurance Plan.

### Sampling

Artists were eligible if they were in their profession for at least two years, can speak English, and deem to be impacted by the COVID-19 pandemic. Since this study was a quality improvement project that aimed to understand the health needs and access to healthcare services at the Centre, artists had to be registered as a client at the Centre. Maximum variation sampling was employed to gather a broad selection of professional backgrounds and experiences.

### Recruitment procedures

The Administrative Coordinator sent an e-mail invitation via a secure, online platform to all clients who have provided consent for email communication. This email provided a summary of the quality improvement project, its goals, duration of participation, and mode of participation (online). Those who are interested were instructed to reach out to the principal investigator of this study (SAL) via email, who responded by enclosing copy of the consent form via email. Clients were asked to reach out again and confirm in writing that they consent to conducting the individual interview with the prinicpal investigator, with full awareness that the interview will be audiorecorded on Microsoft Teams©. Recruitment occurred between February and May 2022.

### Data collection and management

The lead author (SAL) conducted individual interviews using a semi-structured interview guide on Microsoft Teams©. We pilot tested the individual interview guide with two artists to assess the feasibility and clarity of the interview questions. Since none of the questions required modification, data from these interviews were included for analysis. Data collection and analysis occurred concurrently. In keeping with interpretive description [13], data saturation was not the desired outcome as it was considered that there might be an infinite variety of perspectives and experiences when exploring the subjective experiences and meanings. Instead, the focus was to interview informants until a deep understanding (signified by having rich and detailed information about each informant’s context and their perspectives, and reaching and a diversity of perspectives and experiences from different informants) was obtained while recognizing that perspectives deviating from emerged themes may still exist.

Individual interviews were audio recorded and automatically transcribed verbatim (using artificial intelligence) and encrypted by Microsoft Teams©. These transcripts were checked for accuracy and de-identified (names, affiliations, locations were replaced by pseudonyms) by the co-authors of this study (CJ, CS). The de-identified transcripts were uploaded to NVivo 12 [14], a qualitative data management software that facilitated data organization and coding. Interviewer had access to identifying information about the informants during the interview, because informants alluded to the different associations and organizations to which they belonged when they described their experiences on the impact of COVID-19 pandemic on their health and healthcare access. However, this information was removed during the de-identification process once the transcription was made available, and audiorecordings were deleted after de-identification and verification of the transcript’s accuracy.

### Data analysis

Data analysis was an ongoing iterative process conducted by all authors throughout data collection. All transcripts were read in full at least twice, which enabled the identification of similarities and differences between informants, making it possible to observe patterns and generate initial codes. CJ and CS co-developed the initial codebook that contained each code and its definition, with at least one illustrative quote that represented the concept of the code. The codebook was finalized after the 6^th^ interview through two consensus meetings with SAL. Quotes from each interview were assigned to codes. All interviews were coded independently and in duplicate by all authors. Disagreements in codes were resolved through three consensus meetings.

We conducted a secondary data analysis on an existing set of 17 individual interviews that included perspectives on how the pandemic has impacted the health and wellbeing of artists. These interviews occurred between October 2020 and February 2021, and were part of a previous study that aimed to understand the perspectives of artists who received subsidized health services on the barriers to accessing healthcare services. Since the pandemic coincided with the time when these interviews were held, informants spontaneously discussed the impact that the pandemic had on their health and access to healthcare. Findings of this study can be accessed in the published report [15]. Secondary data were also used to confirm the findings of the primary data.

The secondary analysis of these transcripts followed the same analytical methods as primary data. Once the preliminary analysis was completed, we used the codes to develop the themes organized by the analytic framework (SEM). Reviewing of the codes and transcripts were interspersed with strategic periods of immersion in the SEM and performing arts literature as part of the process of synthesizing, theorizing, and conceptualizing in interpretive description [16].

### Maintaining rigour

Several strategies were used to maintain rigour. First, we achieved credibility of our findings by using negative case analysis (actively searching for data that do not support the patterns from the analysis). Second, we included reflection summaries as we made key analytic decisions, which served as an audit trail. Third, we confirmed our interpretations with a random sample of five informants who consented to review a summary list of key findings of this study. We also remained reflexive throughout the research study by reflecting on how our professional backgrounds and preconceived notions may have influenced the interpretation of findings. We completed the Standards for Reporting Qualitative Research Checklist [17] (S1 Appendix) to facilitate methodologic transparency and reporting of research findings.

## Results

### Demographic characteristics

In total, 31 informants were included in the primary and secondary data (Table 1: demographic characteristics). Among these informants, 12 (38.7%) were male, 17 (54.8%) were female, 1 (3.2%) was gender neutral, and 1 (3.2%) preferred not to disclose their gender. The largest category of informants consisted of musicians (n = 12, 35.3%), followed by the second largest category of actors/actresses (n = 7, 20.6%). The proportion of performing artists (n = 25, 73.5%) was larger than that of creative artists (n = 9, 26.5%).

**Table 1:** Demographics of informants.

Characteristic	Primary Data n (%)	Secondary Data n (%)
**Gender**		
**Male**	2 (14.3)	10 (58.5)
**Female**	10 (71.1)	7 (41.2)
**Neutral gender**	1 (7.1)	0 (0)
**Prefer not to disclose**	1 (7.1)	0 (0)
**Total**	14 (100)	17 (100)
**Profession**		
**Musician**	4 (28.6)	6 (35.3)
**Visual Artist**	2 (14.3)	3 (17.7)
**Writer**	1 (7.1)	2 (11.8)
**Actor/Actress**	3 (21.4)	3 (17.7)
**Comedian**	0 (0)	1 (5.9)
**Photographer**	0 (0)	1 (5.9)
**Dancer**	0 (0)	1 (5.9)
**Theatre Worker**	0 (0)	1 (5.9)
**Film Producer**	3 (21.4)	0 (0)
**Arts Educator**	1 (7.1)	0 (0)
**Total**	14 (100)	17 (100)
**Length of time in profession, yrs**		
**0–5**	3 (21.4)	4 (23.6)
**6–10**	2 (14.3)	5 (29.4)
**11–15**	4 (28.6)	2 (11.8)
**15+**	5 (35.7)	6 (35.3)
**Total**	14 (100)	17 (100)

Fourteen artists were individually interviewed for the main study between February 2022 and June 2022. Secondary data analysis was conducted on 17 individual interviews that occurred between October 2020 and February 2021. For those who initially responded to the main study recruitment, 5 (29.41%) respondents had been infected with COVID-19, 9 (52.93%) respondents had not been infected with COVID-19, and 1 (6.2%) respondent was unsure whether they had been infected with COVID-19 or not.

### Transcript pages and interview length

Primary data consisted of 205 interview transcript pages spanning 652 interview minutes. A total of 196 interview transcript pages spanning 637 interview minutes were analyzed for secondary data. The average length of interviews were 47 minutes for the primary and 37 minutes for the secondary data. Overall, the analyses of both types of data consisted of 401 interview transcript pages spanning 1289 interview minutes.

**Fig 1:**
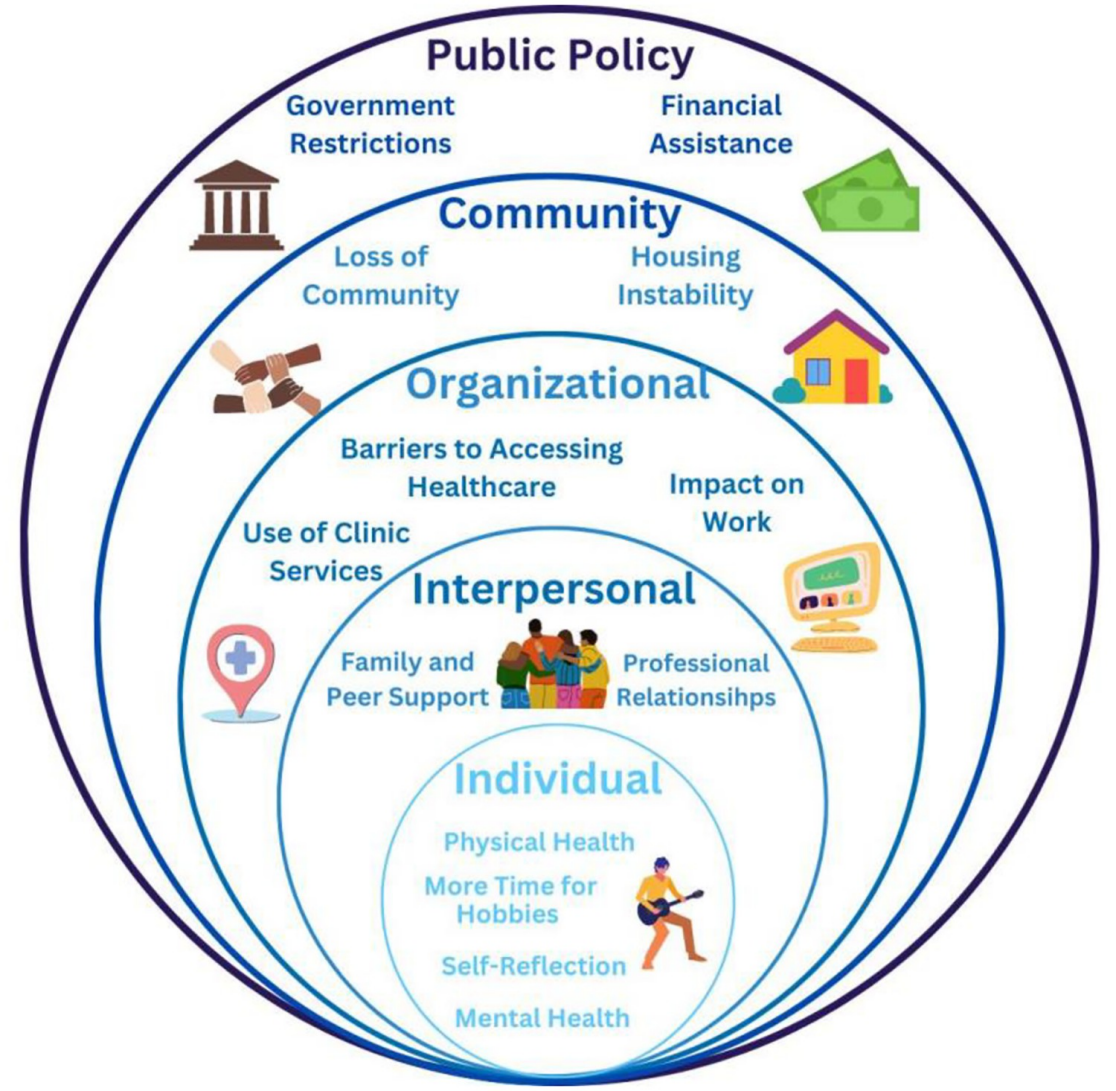
Visualization of factors influencing artistswithin the SEM. This visual representation provides a framework for understanding the multifaceted influences on artists’ health during the COVID-19 pandemic.

The social-ecological model categorizes factors affecting health and healthcare into five levels: public policy, organizational, community, interpersonal, and individual [18]. We explore how these factors, within and across levels, impact artists’ health and access to healthcare during COVID-19 (Fig 1).

### Public policy level

Our study revealed that two public policy-level factors, namely government restrictions and financial assistance, had a significant impact on artists. These individuals, who frequently grappled with precarious employment before the pandemic, experienced particular hardship when the restrictions resulted in the indefinite closure of their studios, theaters, and schools. Furthermore, these public policy-level factors had cascading effects on various aspects at the community level, such as the loss of connection with their communities and challenges in healthcare access, as well as on individual factors, including self-identity and introspection.

Limitations on in-person gatherings had a detrimental effect on artists’ job prospects and opportunities for performances. Consequently, many artists experienced financial hardships due to the lack of work. One informant shared,

My head was hitting the wall many times due to that [pandemic]. I even lost some clients because they were like, ‘well, we want to go back to the regular way of doing things, and we’re going to wait until the government makes a decision and opens things up back normally’. That doesn’t help me because I kind of lost my job.

(Nicholas, Dance Instructor)

In response to widespread job losses as a result of public health restrictions, government-led financial assistance programs such as the Canada Emergency Response Benefit (CERB) were implemented to provide monetary support to artists. Many artists received at least one of these government supports, and most artists reported that the assistance has helped them overcome financial difficulties:

I live in Toronto and my main expense is my apartment. That was a big concern; how am I going to pay my rent every month? I think that without CERB, I honestly don’t even know what would have happened because that was the lifeline for me.

(Thora, Singer-Songwriter)

However, the complex eligibility criteria associated with these programs also posed challenges for many artists in accessing the support they needed. Due to the inconsistencies in funding eligibility, some artists were cautious and confused about the amount they should be claiming or whether they even qualified for the assistance. Two informants shared:

We were lucky that we had CERB, but even that got very scary when they started asking people to send it all back. You also just have to be really careful. I would just be cautious about CERB.

(Lorraine, Writer)

The government is weird because at first; they said artists are gig workers, but they do not qualify for the fund. Then they said that we qualify and now they started cutting it.

(Orianna, Percussionist)

When the sudden loss of government support occurred and government restrictions remained in place, artists were left feeling deeply disappointed and frustrated, as they believed their voices were not being heard. The lack of control over government decisions further exacerbated their already pessimistic outlook on the future:

I felt it was very pessimistic, very disappointing. Of course they’re going to cut [out] the arts first. But also it felt like there was less hope in this situation because, how are we going to survive? Grants are already hard to come by and now, extra supports are going out the door…just hopeless.

(Perri, Oil Painter)

Government restrictions and policies on what was determined as essential workers, prompted some artists to engage in self-reflection regarding their professional identities and the perceived value that they bring to the society. Quintus (Musician) shared, “I don’t think they [society] view us as important, or they view us as auxiliary.” Britney (Film Artist) also expressed her frustration:

If we did a 24-hour no music, no books, no Netflix, no T.V., no turning on Spotify, no nothing. Suddenly everybody would realize that, ‘yeah, art actually gets me through my day’. But somehow we get left out financially and now we can’t afford to live in the city that we made so cool…We always get treated like we are less than or we are taking the easy credit. Do you want to work 65 hours a week for $500? Try it. I just think people don’t really understand at all.

### Community level

In our study, we identified a few community-level factors that had a significant impact on artists, one of which was the profound loss of a professional community that included colleagues, agencies and immediate connections to other resources that would help grow their art careers. The physical isolation resulting from pandemic restrictions not only led to a sense of mental isolation but also prevented the exchange of relevant information about job prospects and connection with key individuals who may lead to career opportunities. For artists, the presence of an artistic community plays a vital role in shaping their personal identity. The sudden disconnection from this community caused many artists to deeply question their identities and core beliefs.

Additionally, artists commonly rely on social connections established in their professional community to find work in their field, all of which were accomplished in-person. One informant explained the challenges of making these connections online, subsequently feeling the impacts of lost work opportunities:

All the events that I ever go to, those are the people that hire me. I get all the contracts I’ve ever gotten through people I have met in person. So, not having access to those venues where I’m usually attending and informally talking to people, it just totally cut out my possibility of finding work. I’ve been going nonstop to panels and to networking events online. But it’s not the same.

(Ivanka, Theatrical Performer)

During the interviews, numerous participants expressed a strong desire for the Centre to facilitate group programming aimed at reconnecting artists with one another. These programs should be easily accessible, because they would provide a valuable opportunity for artists to rebuild their sense of community and establish meaningful connections with their peers.

It could also just be like a bunch of artists getting together and being like,‘this is my life, and this is what I’m doing.’ I think it’s because I just felt so disconnected from the community and [the] people who might understand the specific impact on the type of work I do.

(Carrie, Filmmaker)

Loss of community also sparked deeper reflections on how artists might come together to form a stronger, more resilient community. Ivanka (Theatrical Performer) shared, “*How can we work together as a community to make sure everybody is taken care of*? *We are a community and there has to be a community mobilization*. *I know there’s a lot of organizations that offer support*, *but it’ll be so good to have artists coming together and banding together to help each other out*”.

Informants also suggested that the Centre host virtual spaces to create more opportunities for restoring community: *“I’d be curious to know if there were any*… *Zoom groups that you could sign up for*… *Maybe a therapist could lead a session or meditation*… *That would be cool to know that there are services that create more community*.*”* (Lorraine, Writer) Another suggested the idea of having the Centre “*offer space for people to do stretch classes of their own or*, *or for people to do some kind of a community engagement; maybe just a space to offer people to circulate skills or talents*” (Nicholas, Dance Instructor).

### Organizational level

At the organizational level, our study revealed that artists experienced significant impacts on their work and access to healthcare services, including general care and specialized support from the Centre, due to COVID-19. Organizational-level impacts of the pandemic prompted some artists to reconsider their career paths. Faced with uncertain prospects and limited opportunities, some artists found it necessary to explore alternative avenues or diversify their skills outside their traditional artistic disciplines. One informant shared,

The most heartbreaking thing is seeing people make a huge pivot into career paths that they never really wanted to go down on because they needed to. Very early on in the pandemic there was a lot of discourse in the artist community saying this [pandemic] isn’t going to pass quickly. It is advised that you lean into whatever side jobs you’re doing, whether it’s video editing or graphic design. The full-time freelancers are hit the hardest because all of a sudden they had nothing in their calendars and some decided to pick up a job at Loblaws. Many of my friends have moved out of Toronto to save a little bit on rent. Some of my friends had a full career change because they don’t want to wait for this to be over.

(Chris, Musician).

Furthermore, the pivot to online platforms as a substitute for in-person engagements was not uniformly feasible for all artists. The access to and proficiency in utilizing digital tools and online platforms varied, creating disparities among artists in their ability to adapt and continue their artistic practices. One informant shared, “I pivoted to virtual and people did not really know how to do virtual well.” (Myla, Writer)

Artists who transitioned to administrative work to cover their living costs as a result of pandemic restrictions, could not access healthcare services as readily as before. One informant shared, “*I was having such a hard time accessing health services in general because I work nine to five now*, *and so many things are only open nine to five*, *including the Center*. *I just can’t get away from work to access these services*”. (Thora, Singer-Songwriter)

During the height of COVID-19, artists reportedly encountered multiple barriers to accessing healthcare needs, akin to those faced by individuals in low-income neighborhoods, as illustrated in the following quote:

There’s definitely a lot of barriers to access at multiple different levels for healthcare needs. I think we [artists] go through many of the same problems of low-income neighborhoods. We’re starting to see those kinds of cracks [in the healthcare system]; people who are suffering from the effects or deaths of COVID-19…the numbers are much higher in low-income households. We [artists] share some of those similar barriers and issues because we are also in a very low-income bracket.

(Carmen, Musician).

The closure or transition of in-person clinical services to online platforms posed challenges for artists in accessing their regular healthcare. While counselling and physiotherapy sessions could more readily adapt to virtual delivery methods, other forms of care, such as massage therapy or acupuncture, had to be postponed until public health restrictions were lifted. Even with virtual options offered for some services, some informants noted differences in the virtual care compared to in-person visits: “*For me*, *physiotherapy needs to be hands-on*. *I mean*, *it’s great video stuff*, *but I actually need the hands-on*.” (Unna, Musician). Another informant shared, “*Certainly it [counselling] isn’t the same as being across from a human being*. *But I’m grateful that I’ve been able to continue with them [psychotherapist] and similarly with the online cranio-sacral appointments*. *The treatment provided was just not the same as it would have been in person*. *But I was still very grateful to have it*” (Marie, Writer).

One informant provided a compelling perspective on the importance of having a healthcare clinic that understands the unique challenges faced by artists. She emphasized the difficulties of fitting into traditional societal categories and how having a clinic that understands these intricacies removes significant barriers to allow for a deeper understanding of the intersections between their artistic profession, wellbeing, and financial stability:

We [artists] don’t fit into little boxes because we’re so special and precious; we have a weird career and it doesn’t fit [into societal categories]. And not having to compress that—having that understood—removes a massive barrier. Having a clinic that basically understands the intersections of [artistic] profession…You know, precarious incomes are super common, but I can’t explain that to my bank, right? There’s an understanding [at the Centre] what the shape of our lives is, and moving to meet us halfway, is incredibly valuable.

(Myla, Writer)

### Interpersonal level

At the interpersonal level, artists shared about their relationship dynamics in relation to their colleagues, families and friends. For many artists, their lives revolved around spending time with others, making the sudden disconnect all the more difficult. Wrenn (Actor) shared, “*I am definitely an extrovert*, *and [COVID] took a tremendous toll on me not being able to see people and gather*… *Interacting with others is an integral part of how I navigate the world*, *so being alone was incredibly tough*”.

With the advent of the pandemic, widespread social isolation became a reality for most people, resulting in a loss of social connections. Artists were particularly affected due to the highly social nature of their careers before the pandemic. One artist expressed the impact of this disconnection by saying, “*My life is very social*, *and my social life was very much intertwined with the music industry*. *So*, *all those people were like family to me*… *and I feel like I’ve lost that*, *which has been incredibly challenging*” (Thora, Singer-Songwriter).

Artists who had the support of close family members found comfort and solace during this time. Living with family members provided a sense of emotional support and reassurance. For instance, Perri (Oil Painter) expressed that she stressed about finances during the pandemic but felt comforted: *“Knowing that my family will try to help me if I have problems*. *Like if it gets too bad*, *at least there will be someone there that I can fall back on*.*”*

However, other artists felt more isolated from their families during the pandemic. Carrie (Filmmaker) did not have family members in the arts so she did not know where to go for support during the lockdown:

Nobody in my family has ever worked in the arts. I think that also increased the isolation I felt in my experience because I didn’t really have anyone to help reassure me or guide me… So, there is a lot of questioning if I had made the right decision to do this and if I should be doing something else.

Lack of support from family also prompted some informants to suggest support groups that could be organized by the Centre:

A lot of artists have lovely families, but there’s a disconnect…Often, families always come back to the point of, ‘It’s all your fault. You know you’ll never get out of this if you stay an artist’. So getting artists together to discuss those kinds of things in a conversational mode…that’s genuinely vital to the wellbeing of everybody.

(Ivanka, Theatrical Performer)

### Individual level

At the individual level, our study revealed significant impacts on artists’ health and newfound opportunities for hobbies and self-reflection due to the pandemic restrictions. The restrictions and resulting isolation caused a notable increase in anxiety and depressive symptoms among many of the informants. Financial losses and work disruptions were identified as major stressors, with many artists emphasizing financial insecurity as one of the main challenges during the pandemic. One artist articulated her fear about financial uncertainty: “*I think it’s just the fear of what happens next*. *As artists we’re used to having financial worries*. *You do your best to have yourself set up financially*. *But that just wasn’t possible this time around*. *I’m lucky that I had some inheritance and some savings that I could live on*. *But I’m at the end of that*.” (Lorraine, Writer)

The uncertainty and disruption in their professional lives had detrimental effects on how artists viewed themselves. Some informants highlighted the central role of their work in shaping their identity and sense of purpose. Not being able to pursue their artistic endeavors during the pandemic left them feeling lost and struggling with a sense of purpose. Carrie (Filmmaker) admitted, *“I just felt a little bit lost*. *I wasn’t sure what I was going to do*. *I think work has been something that has affected my conception of myself or my identity… I struggled feeling a sense of purpose*.”

The association of work with self-worth was also evident, as one informant shared feelings of inadequacy due to the lack of productivity caused by the restrictions:

Self-worth…our value is completely tied to our productivity, which is the capital we produce. Thus, in the stages, especially in the beginning, where you were completely locked down, it was very difficult to do anything. I think I struggled with feelings of inadequacy and not being productive.

(Amanda, Violist)

However, the additional time afforded by the restrictions presented an opportunity for artists to engage in activities they had previously been too occupied to pursue. Some artists reported using this time for regular exercise, improving their physical well-being. The increased availability for self-reflection also allowed artists to evaluate their work-life balance and prioritize aspects that were of utmost importance to them. This introspection led to profound realizations, as expressed by one artist who discovered the importance of finding reassurance within oneself:

And what is the greatest source of reassurance or comfort during the pandemic? It’s so interesting. I think it’s myself. I think it’s the time. Because the job of an artist is, you’re just hustling all the time and you’re running from gig to gig and opportunity to opportunity. And you’re just chasing this dream. When everything stopped, it really allowed me to look and ask, ‘Is that a healthy lifestyle that I can keep up?’ It gave me an opportunity to reflect.

(Lorraine, Writer)

Some informants had a significant realization that their identity had become excessively tied to being an artist, leaving them unsure of their purpose beyond artistic pursuits. They articulated the artist’s profound identification with their artistic role, to the extent that other aspects of their life were neglected or given less priority. Losing work under these circumstances intensified their fear and uncertainty. This realization prompted the artists to embark on a personal journey of self-discovery and re-evaluation, as they sought to redefine themselves beyond their artistic identity. For instance:

[It was a] really rude awakening that once I lost my job in 2020, I really realized that I identified so much as an artist, that I’d let other parts of my life go or lose priority. And it was quite horrifying and humbling to realize that I’d almost over-identified as an artist and didn’t know what I did for myself anymore. That was what made losing work so doubly scary. I think a lot of people felt, ‘oh, if I’m not working, [then] who am I like? I don’t know what I am outside of being this.’ So that was definitely something that I worked on a lot and am still working on.

(Thora, Singer-Songwriter)

## Discussion

In their scoping review of 21 studies on the impact of COVID-19 on performing artists [19], Brooks & Patel (2022) identified several major themes. These themes included job loss, financial instability, loss of social connections, uncertainty about the future, and impact on mental health and self-identity; all of which were also identified in our study. This indicates that Canadian artists share similar experiences with artists in other nations including Germany, Belgium, Australia, England, China, and the United States (US). Using the SEM allowed us to take these themes one step further and begin to explore how various factors at different levels—including individual, interpersonal, organizational, community, and public policy—interact and shape artists’ health and their livelihoods.

Our findings demonstrated the significant impact of public health restrictions and the availability of financial assistance programs at the public policy level on multiple aspects of artists’ lives. These factors directly influenced the stability of artists’ housing, their professional community, and their access to essential resources at the community level. Artists in our study recognized that government assistance programs played a crucial role in helping them cope with the pandemic-induced financial hardships; however, eligibility criteria and the application process for these programs were unclear, which has been also reflected in the United Kingdom (UK), US, and Australia [19–21]. Artists in our study felt unsupported and undervalued by the government while public health restrictions were in effect, which mirrored the sentiments of performing [9, 22] and creative artists [23] in the UK and Australia, respectively. Furthermore, the decision of whether an artist transitions to non-artistic jobs during the pandemic was not only triggered by these public policy factors but also by a broader range of factors in other levels, including input from their social networks within the artistic community (community level).

The unique combination of these different factors at various levels within the SEM have an impact on individual-level outcomes, including the artist’s mental health status and perceptions on self-identity. Unlike individuals in other professions, for whom work and self-identity tend to be separate, artists experience a strong interconnectedness between the two. The impact of the pandemic on self-identity was a shared experience between the artists we interviewed and the performing artists in the UK [9, 22]. Art, creativity, and working in the arts were seen as central to the our study informants’ identities and perceived to be tied to their self-worth; in turn, the reduction in artistic pursuits during lockdown led to a loss of self-worth. Creative artists in Australia also shared similar perspectives [23]. The long-term effects of restrictions, as identified by Wright and her team [24], have been shown to have detrimental impacts on productivity and decision-making. Our findings align with previous research by Spiro et al. [9] whose participants also reported feelings of anxiety, uncertainty and concerns about progression in their artistic career during the pandemic. This extends beyond the artistic community, as extensive research consistently demonstrates the association between public health restrictions and elevated levels of depression and anxiety at the individual level [8, 9, 19, 21, 25].

Although the general population also experienced high levels of uncertainty, most individuals were able to maintain employment and establish daily routines [20], unlike many artists. Artists who are already in lower economic positions found that the pandemic exacerbated their financial challenges. This inability to adapt financially forced some artists to switch to less fulfilling jobs, as observed by Stuckey et al. [26]. The artists we interviewed expressed dissatisfaction with having to take on non-artistic work to sustain themselves, which further impacted their mental health alongside the overall stress of the pandemic. Contrary to studies focusing on the general population that often presented a positive perspective on the shift to virtual employment [20, 27], our findings demonstrated the additional burdens faced by artists.

One crucial protective factor against these mental health challenges identified in our study was the significance of social support at the interpersonal level in enhancing mental well-being during the pandemic. This finding aligns with the research conducted by Gaspar et al. [25], which emphasizes the importance of protective factors such as spending time with family and friends. On the contrary, O’Sullivan et al. [28] found a higher prevalence of severe loneliness during the pandemic among those lacking interpersonal relationships and a sense of community. As observed in our study, the loss of social interactions within their professional community extends beyond the individual health effects; this also impedes networking opportunities, as noted by Wright et al. [24], leading to a noticeable reduction in job opportunities at the organizational level.

At the organizational level, some of the artists we interviewed vocalized the challenges of accessing critical, self-funded healthcare services such as psychotherapy, counselling and physiotherapy. Exacerbated by the pandemic, many artists suffering from mental health challenges do not have the financial means to access mental health services. Offering more subsidies to artists in financial need may help with facilitating healthcare access to get the timely treatment they need, as demonstrated by Li et al. [15]. Equally important, artists, as exemplified in our study, belong to a unique community that has inherently distinct challenges to their mental health during times of crisis. In light of the complex circumstances and precarious employment positions often faced by artists, mental health interventions should be designed with careful consideration. Rajkumar et al. [8] suggest the implementation of targeted interventions that address the specific mental health concerns of distinct populations in the aftermath of COVID-19.

Since the pandemic, policy responses across nations have increased efforts to promote recovery in the arts industry. In Australia, several associations have offered grants and resilience funds [23]. In Canada, the creative and performing arts industries have received a large injection of funding to help the arts community to transition their work to digital platforms [29]. Various charities and non-profit organisations in the US have also provided emergency relief for artists [30]. Although these programs may alleviate immediate financial burdens, they are unlikely to address long-term financial impacts of the pandemic [9].

To our knowledge, this study is the first to apply the SEM as an analytical framework to understand the impact of COVID-19 on artists’ health. Applying SEM as an analytical framework holds tremendous potential for advancing our knowledge base in this field. By achieving consistency across studies in different contexts, this approach facilitates the identification of patterns, trends, and similarities across different artist populations, ultimately achieving a comprehensive understanding of the complex interactions between factors influencing artists’ health. This approach helps researchers recognize that artists’ health outcomes are influenced by a combination of individual characteristics, social relationships, community resources, and broader societal structures. Organizing data using the SEM aids in the development of targeted and effective interventions. By understanding the various levels of influence and their interactions, researchers and policymakers can design interventions that address multiple determinants simultaneously, taking a multi-pronged and holistic approach to sustainable change.

Researchers can explore how interventions addressing one level of influence can have positive effects on other levels. For instance, an intervention that enhances social support networks and community resources (interpersonal and community levels) may also lead to improved mental health outcomes for individual artists (individual level). Acknowledging that artists’ interpersonal relationships significantly impact their well-being, may inspire the development of diverse and inclusive peer support networks, ensuring that artists can connect with others who share similar experiences or challenges, fostering a sense of community. Artistic communities often act as crucibles for creativity, resilience, and mutual support, amplifying the protective factors that contribute to the well-being of individual artists. By recognizing the importance of preserving physical spaces that encourage community building, governments and non-profit organizations can help in securing infrastructure that serve as the physical embodiment of the artistic community. The provision and preservation of such spaces can both contribute to flourishing creativity but also stand as tangible safeguards for the sense of belonging that underpins the mental health resilience of artists. Recognizing the intricate connections between levels of the SEM can guide the development of interventions that have broader and more sustainable impacts on artists’ health.

### Limitations

Readers should consider the following limitations when interpreting study findings. Participant recruitment and interviews were conducted virtually using an online platform, which required informants to possess technological literacy, access to a computer or smart device, and an internet connection. Notably, informants were sampled exclusively from a single health center in Toronto, Canada, and therefore may not represent the diverse range of experiences among Canadian artists across different geographical areas. Furthermore, the timeframe of our research focused on capturing the experiences of artists from pandemic onset to a point closer to its end. Consequently, our results and discussion did not have the opportunity to fully understand the artists’ transition out of the pandemic. Secondary data analysis presents certain limitations as the data were not originally collected to address the research question of our present study. Notably, the primary data we gathered demonstrate consistency with the secondary data, thereby supporting the credibility and appropriateness of the secondary sources.

## Conclusions

The long-term effects of restrictions during the pandemic have had detrimental impacts on their professional community, mental health, and employment opportunities for artists. The sudden loss of employment precipitated by the restrictions hindered their ability to swiftly recover. It is imperative for government assistance programs to extend their support beyond the immediate aftermath of restrictions, allowing individuals the necessary space and resources to rebuild their art. While these programs should not be expected to provide prolonged financial assistance, they should incorporate a transitional period to facilitate readjustment. Noteworthy is the revelation that many artists, through government financial assistance during the pandemic, surpassed their annual earnings from artistic endeavors. This highlgihts the need for targeted interventions and support mechanisms tailored to the precariousness of their employment. The COVID-19 pandemic has brought into focus the fragility of the social determinants of health for artists, accentuating the importance for interventions and policies that address the unique challenges inherent in their profession.

## Supporting information

S1 Appendix Standards for Reporting Qualitative Research (SRQR) checklist. A checklist with indexed page numbers indicating how the manuscript fulfills the SRQR criteria.
(DOCX)
